# The phenology of winter rye in Poland: an analysis of long-term experimental data

**DOI:** 10.1007/s00484-015-1127-2

**Published:** 2016-01-05

**Authors:** Andrzej Blecharczyk, Zuzanna Sawinska, Irena Małecka, Tim H. Sparks, Piotr Tryjanowski

**Affiliations:** 1 Department of Agronomy, Poznań University of Life Sciences, Dojazd 11, 60-632 Poznań, Poland; 2 Institute of Zoology, Poznań University of Life Sciences, Wojska Polskiego 71 C, 60-625 Poznań, Poland; 3 Sigma & Faculty of Engineering, Environment and Computing, Coventry University, Priory Street, Coventry, CV1 5FB UK

**Keywords:** Agricultural practices, Crop phenology, Long-term experiment, Monoculture, Winter rye, Yield

## Abstract

The study of the phenology of crops, although quite popular, has limitations, mainly because of frequent changes to crop varieties and management practices. Here, we present data on the phenology and yield of winter rye in western Poland collected between 1957 and 2012 from a long-term field experiment. Data were examined for trends through time and compared to climatological factors using regression analysis. Both annual air temperature and precipitation increased during the study period, equivalent to 2 °C and 186 mm, respectively, over the 52-year period for which met data were available. We detected significant delays in sowing date and recently in emergence, but significant advances were apparent in full flowering date equivalent to 4 days/decade. Yield and plant density experienced a step like change in 1986; yield increasing by ca. 70 % and plant density increasing by ca. 50 %, almost coinciding with a similar change in annual mean temperature, but most likely caused by a changed seed rate and use of herbicides. Future climate change is expected to have a greater impact on this crop, but farmers may be able to adapt to these changes by modifying water regimes, using new machinery and sowing new rye varieties.

## Introduction

The observation and recording of phenological events has a long tradition. This tradition is closely related to the observation of weather and a dependency of mankind on the seasons. Phenology is especially important for understanding the development of cultivated plants (Chmielewski et al. 2004; Menzel et al. 2006a). Phenology was initially defined as “the study of the timing of recurring events, the causes of their timing with regard to biotic and abiotic forces, and the interrelation among phases of the same or different species” (Lieth 1974). More recent definitions, however, stress the important influence of the environment; Schwartz (1999) notes that “phenology includes the study of periodic events as influenced by the environment, especially temperature changes driven by weather and climate.” Phenological dynamics are determined by complex interactions between genetic and environmental factors. Among the plant phenological phases, flowering time is the most frequently reported, because it is one of the simplest to record and one of the easiest to interpret (Chmielewski et al. 2004; Menzel et al. 2006a). Researchers have found air temperature to be a dominant factor controlling the timing of flowering and other phenological phases (Hunter and Lechowicz 1992; Galán et al. 2001).

The timing of when farmers begin to sow and harvest crops varies from year to year and is influenced by weather. Based on long-term observations of these events, practical recommendations have been provided to farmers in the form of phenological calendars (Chmielewski et al. 2004; Menzel et al. 2006a). Calendars have been compiled for many wild and cultivated plants (Chmielewski et al. 2004; Menzel et al*.*2006a), although recent trends in phenology may result in calendars now being inaccurate (Wittchen and Chmielewski 2005).

Among the crops most influenced by climate factors (Wittchen and Chmielewski 2005) is winter rye (*Secale cereale* L.). The Food and Agricultural Organization of the United Nations (FAO, www.faostat.fao.org) reported that world production of rye in the year 2011 was 12.9 million tons, of which three countries contributed 62 % (Russian Federation 23 %, Poland 20 %, Germany 19 %). Rye grows well in much poorer soils than those required for most other cereal crops. Thus, it is an especially valuable crop in regions where the soil is sand or peat (Barnes and Putnam 1986; Schlegel 2006). Furthermore, rye will survive snow cover that would kill winter wheat (Prończuk et al. 2003).

The phenology of winter rye is not just important from a food production perspective but also with regard to pollen and allergens produced by the crop (Barnes and Putnam 1986). Within a region, the relative abundance of different pollen-producing plant species, their number of flowers and inflorescences, anther productivity, weather conditions, and abiotic factors all contribute to determine the pollen load in the air and thus its potentially allergenic effect (Myszkowska et al*.*^2011^). In Poland, winter rye is considered to be a major allergen (Myszkowska et al. 2011; Kruczek and Puc 2012). Analysis of phenological data is thus also important to generate a regional pollen calendar and to predict the periods of increased pollen counts, which are important to know for the treatment of pollinosis (Kruczek and Puc 2012).

Making information available for farmers and medical services is sometimes difficult, especially for cultivated plants, because long-term series are distorted by changes in management regimes, sown varieties and soil nutrients (Chmielewski and Köhn 2000; Williams and Abberton 2004). Management by farmers is adapted not only to climate but also to local market prices and to technological advances (Chmielewski et al. 2004). Therefore, making long-term analyses of crop phenology is not trivial (Chmielewski and Köhn 2000; Williams and Abberton 2004).

Here, we present analyses on long-term data on winter rye in western Poland. We analyzed phenological changes over a 55-year period, the relationships between different phenophases, and the relationship of phenology to climate factors. Finally, we discuss the effects of changes in management practices, sometimes called farmer adaptations (Wittchen and Chmielewski 2005; Menzel et al. 2006a).

## Materials and methods

Our study is based on a long-term experiment established in 1957 whose original aim was to demonstrate how fertilizers affected soil fertility and the yield of winter rye. The experiment was at the Brody Experimental Station (52° 26′ N, 16° 18′ E, 92 m a.s.l.) of the Agronomy Department of Poznań University of Life Sciences, located 50 km west of Poznań in the Wielkopolska Region of western Poland. It was established as a randomized block design of four replicates on a podzolic soil. The soil texture was sandy loam with underlying loams. Prior to the experiment, the soil had a pH of about 6.0 (pH in mol KCl dm^−3^), a very high phosphorus content, average magnesium and potassium content, and contained about 7 g kg^−1^ of organic carbon. Fertilizer application has remained fairly constant using a 90:60:120 N/P_2_O_5_/K_2_O compound. In 1986, herbicides were used for the first time. Harvesting methods have also changed over the years; until 1976 using a sheaf-binder, and later using three types of combine harvester: Hege 125 B (1977–1998), Wintersteiger Nurserymaster Elite (1999–2007), and Wintersteiger Classic (2008–). Three varieties have been grown: Wielkopolskie (until 1964), Smolickie (1964/1965–1971), Dańkowskie Złote (1971/1972–2012). Winter rye has been grown continuously since 1957 on the same plots. This is one of the oldest experiments in Poland, and one of only three designed in a similar way in Europe, based on a valid statistical design.

Phenological observations on rye in this experiment have been carried out for 55 years (1957/1958–2011/2012) using the BBCH scale (Lancashire et al. 1991). The BBCH scales are well known worldwide and are used for research, agronomy, and in phenology. In this study, we analyzed the following seven development stages of rye: sowing date (BBCH 00); emergence—leaf not completely unfolded (BBCH 10); stem elongation—start of the growing season in spring (BBCH 30); heading—half of inflorescences emerged (BBCH 55); first flowering (BBCH 60); full flowering—50 % of anthers mature (BBCH 65); harvest (BBCH 89–92). All dates were converted to day of the year (DOY, 1 = January 1, etc.) prior to analysis, with autumn dates (e.g., sowing and emergence) as negative days of the year.

Mean annual winter rye yield data were available for the years 1958–2012. Established plant density in plants/m^2^, average number of grains/ear and fertilizer applications (kg/ha) were available for the years 1972–2012. Further details of the study are discussed in detail in Blecharczyk (1999) and Szajdak et al. (2004). Mean monthly temperatures and monthly precipitation, collected according to WMO guidelines for the years 1961–2012, were obtained from the Brody Experimental Station meteorological station.

Standard regression and correlation methods were used to examine for trends over time, to compare phenological events with each other and with temperature and precipitation. Regression coefficients are presented ± SE. Because three different varieties have been used in this experiment which may have influenced phenology and agronomic variables (e.g., preliminary analysis suggested that mean full flowering dates differed by 13 days between the three sown varieties), analysis has also been repeated using just the data from 1972 to 2012 (single variety in use) incorporating a dummy variable reflecting the seed rate/herbicide change in 1986. Consideration was also given to removing the effect of harvesting/combine type on crop yield. However, a one-way ANOVA testing harvesting method after elimination of seed rate/herbicide change revealed no significant difference on yield (*F*_3,50_ = 1.42, *P* = 0.249). The influence of temperature and precipitation on phenology was investigated by stepwise regression with potential predictor variables being monthly mean temperature and monthly precipitation sum for the 3 months leading up to the mean date of the phase. The seed rate/herbicide change dummy variable was forced into these models to account for any effect of this change on phenology.

## Results

**Fig. 1:**
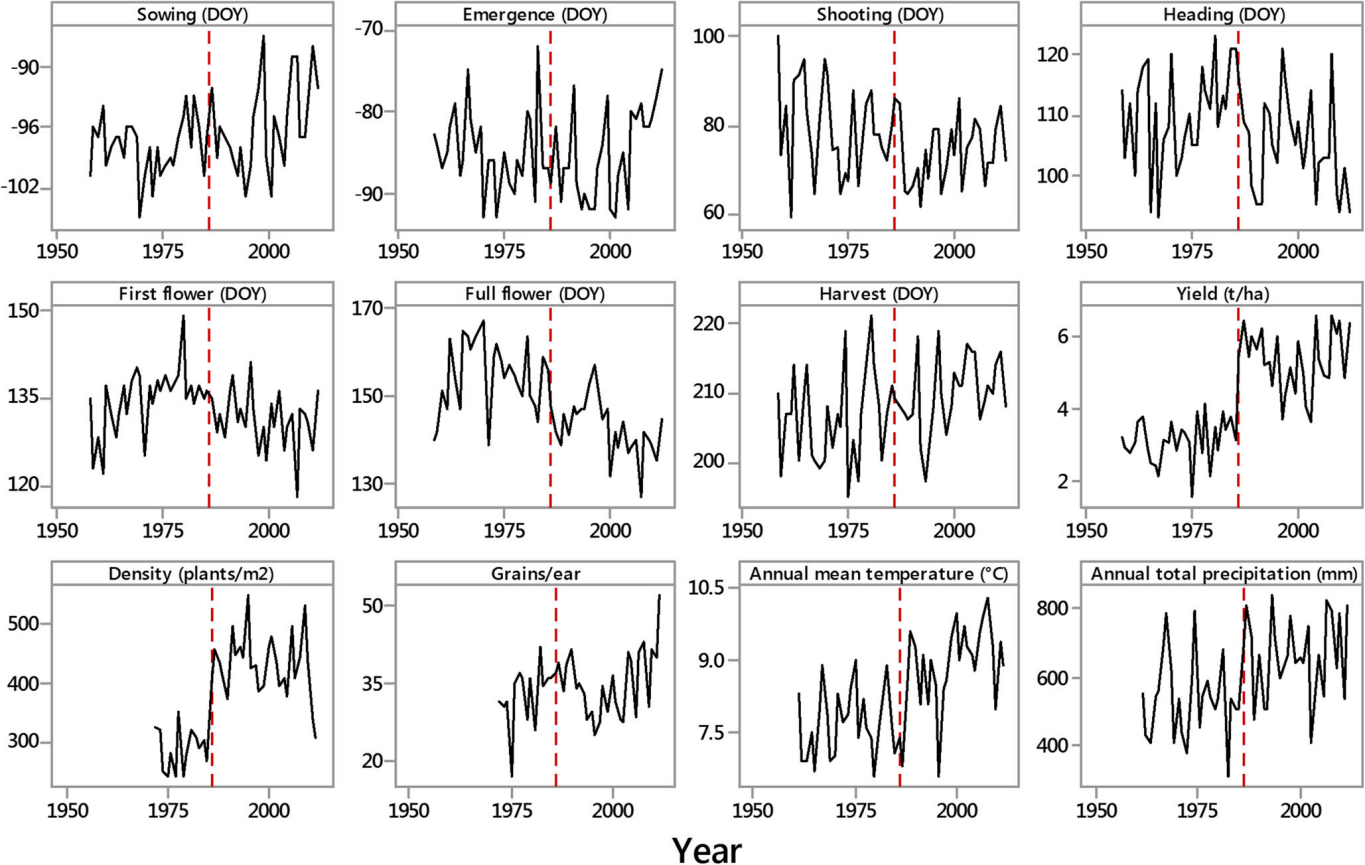
Plots against year of the seven phenological measures in winter rye (day of year (DOY), 1 = January 1, etc.), yield, plant density, grains/ear, annual mean temperature and annual total rainfall recorded at Brody, western Poland. The *vertical line* is at 1986, the year when sowing techniques changed and herbicides were first used. Phenology and yield based on the years 1957/1958–2012, density and grains 1972–2012, and met data 1961–2012

The effect of the seed rate/herbicide change in 1986 was very evident on increased plant density (433 ± 10 in 1986–2012 vs. 287 ± 9 plants/m^2^ in 1958–1985) and yield (5.35 ± 0.16 vs. 3.09 ± 0.11 t/ha, respectively). Mean values of phenological, agronomic, and meteorological information and their trends over time are summarized in Table 1. These data, uncorrected for any change in variety or management practice, revealed significant delays in sowing (by a total of 5 days over the 55-year period), significant advances in shooting equivalent to 9 days and in full flowering equivalent to 20 days, and a significant delay in harvest equivalent to 8 days. There was a significant increase in yield, in plant density, and in number of grains/ear. Both annual temperature and precipitation had increased, equivalent to 2 °C and 186 mm, respectively, over the 52-year period for which records exist. These patterns are shown in Fig. 1. Yield and plant density experienced a step-like change in 1986 likely primarily due to seed rate change and herbicide usage; yield increasing by ca. 70 % and plant density increasing by ca. 50 %, almost coinciding with a step-like change in annual mean temperature after 1987 (the step change model for temperature was a better fit than a linear regression, *R*^2^ 55.9 % compared to 40.0 %).

**Table 1:** Details of the phenological, agronomic, and climatological data of the experiment, together with a summary of linear regression of selected variables over time (regression on year)

	1958–2012								1972–2012			
	Mean	SD	Min	Max	*b*	SE	*R* ^2^	*P*	*b*	SE	*R* ^2a^	*P* ^a^
Sowing	−97 (Sep. 25)	4	−105	−87	0.087	0.030	13.6	0.006	0.225	0.085	20.8	0.012
Emergence	−85 (Oct. 7)	5	−93	−72	0.021	0.044	0.4	0.639	0.358	0.114	20.7	0.003
Shooting	76 (Mar. 16)	9	59	100	−0.167	0.079	7.8	0.039	0.215	0.174	5.3	0.224
Heading	108 (Apr. 18)	8	93	123	−0.127	0.069	6.0	0.071	−0.104	0.176	17.8	0.556
First flower	133 (May 13)	5	118	149	−0.064	0.046	3.5	0.169	−0.152	0.103	33.2	0.267
Full flower	149 (May 29)	10	127	167	−0.363	0.065	37.1	<0.001	−0.398	0.131	57.4	0.004
Harvest	208 (Jul. 27)	6	195	221	0.138	0.050	12.4	0.008	0.251	0.140	10.0	0.081
Yield (t/ha)	4.20	1.36	1.55	6.57	0.064	0.008	56.9	<0.001	0.007	0.019	62.5	0.720
Density (plants/m^2^)	383	85	242	551	4.39	0.89	38.3	<0.001	−1.27	1.12	69.0	0.264
Grains/ear	34	6	17	52	0.163	0.080	9.6	0.049	0.260	0.142	11.2	0.074
Annual mean temperature (°C)	8.3	1.0	6.6	10.3	0.041	0.007	40.0	<0.001				
Annual total precipitation mm	605	131	313	841	3.58	1.11	17.1	0.002				

*b* represents the per year trend which is presented together with its standard error, % variance accounted for (*R* ^2^), and an indication of statistical significance (*P*). Phenological and yield data for 1958–2012, other agronomic data for 1972–2012, and climatological data for 1961–2012

The regression models for 1972–2012 involve just a single variety in use and include a dummy variable (see text for details)

^a^ *R* ^2^ indicates the overall model, but *P* just the significance of the trend through time

Reanalysis of data just for the 1972–2012 period (one variety in use), incorporating a dummy variable to remove the effect of the seed rate/herbicide change, confirmed the delayed sowing date (2.2 days/decade) and advance in full flowering (4.0 days/decade). However, trends in shooting date, harvest date, yield, plant density, and grains/ear were no longer significant. Furthermore, there was also a significant delay in emergence date of 3.6 days/decade.

Of the 21 correlations between the seven phenological phases, eight were statistically significant (Table 2) and the bulk of these were with adjacent phases. The strongest correlations were between sowing and emergence dates, and between first and full flowering dates. There were significant negative correlations between sowing date and the two flowering phases, i.e., late sowing was associated with earlier flowering.

**Table 2:** Pearson correlations (*r*) between winter rye phenophases over 55 years in western Poland

	Sowing	Emergence	Shooting	Heading	First flower	Full flower
Emergence	0.644***					
Shooting	−0.109	0.076				
Heading	−0.227	−0.178	0.315*			
First flower	−0.299*	−0.017	0.303*	0.445**		
Full flower	−0.376**	−0.094	0.213	0.335*	0.671***	
Harvest	0.117	0.007	0.216	0.035	0.117	−0.153

Significant correlations: *P < 0.05, **P < 0.01, ***P < 0.001

Stepwise regression was performed to identify the influence of temperature and precipitation (in the 3 months leading up to the mean of that phase) on the seven winter rye phenophases. Analysis was restricted to the post-1972 single variety stage of the experiment and a dummy variable for change in seed rate/herbicide was forced into each regression. No significant effects of temperature or precipitation were detected on sowing date, emergence date, or harvest date. Negative temperature effects dominated the other phases with a 1 °C rise in temperature associated with a 1.7- to 3.8-day advance in phenology (Table 3). These values are consistent with those found elsewhere (Estrella et al. 2007). Precipitation featured in only one of the models (first flower) and suggested earlier first flowering after wetter Mays. Models of flowering had notably larger values of % variance explained.

**Table 3:** Summary of stepwise regression analysis of winter rye phenophases in 1972–2012 on the mean temperature (T) and precipitation (P) in the 3 months preceding the mean of the event

	Variables selected				T effect	*R* ^2^	*P* value
Shooting	Feb. T	Mar. T					
	−0.95	−1.64			−2.59	48.3	<0.001
Heading	Mar. T						
	−1.65				−1.65	32.7	<0.001
First flower	Mar. T	Apr. T	May T	May P			
	−0.67	−1.14	−0.95	−0.042	−2.76	61.7	<0.001
Full flower	Apr. T	May T					
	−2.40	−1.35			−3.75	68.7	<0.001

The dummy variable for seed rate/herbicide change was forced into each model. No significant model was found for sowing date, emergence date or harvest date. Variables selected indicate the significant terms included in the model with coefficients below variable names. The column headed *T effect* indicates the net effect in days of a 1 °C rise in temperature on that phase

## Discussion

In this study, we present analysis of observational records of seven phenophases of winter rye in western Poland over more than 50 years. In general, adjacent phenophases were positively correlated, which is common in phenological traits (Roetzer et al. 2000; Ziello et al. 2012). This pattern was similar in both our whole time series and in just the last four decades when only one variety was grown (results not shown). However, only some of the phenophases were strongly related to climate events, mainly to temperature, which is in slight contrast to a previous study in Germany (Chmielewski et al. 2004). Obviously, part of the long-term change may be masked by other changes to management techniques (Chmielewski et al. 2004; Menzel et al. 2006b). It is especially interesting that the two phenophases more directly related to human activity (sowing and harvesting) were delayed over time, the former significantly, suggesting an adaptation by farmers, possibly to warmer late summers and autumns (Chmielewski et al*.*^2004^; Menzel et al. 2006b). Obviously, adaptation not only involves the time of sowing and/or harvesting but also the use of different management techniques, and changes in technologies used to sow and harvest. Even changes in varieties have an influence (Howden et al. 2007); however, in our dataset, this effect was not obvious. In consequence, there have been marked long-term changes in crop density and yield, and in the use of artificial fertilizers (Sadowski and Krześlak 1995; Blecharczyk et al. 2004; Wittchen and Chmielewski 2005), although the latter was relatively stable in our study.

Even taking into account farmers’ changes in timing of sowing, crop flowering remains strongly influenced by temperature (see also Estrella et al. 2007), and advanced flowering was noted for full flowering date but not first flowering date. This fact is of concern since it implies not only an earlier peak in the pollen season but a reduction in the interval between first and full flowering suggesting a greater pollen load over a shorter time span. This is important for constructing calendars for allergy sufferers (Myszkowska et al. 2011; Kruczek and Puc 2012; Ziello et al. 2012) and supports the view that these calendars should be changed over time (Ziello et al. 2012). A similar shortening of the flowering period was reported for native and exotic species by Bock et al. (2014). Temperature responses in rye were similar to, if somewhat smaller, that responses of native and exotic plant species recorded elsewhere in western Poland (Sparks et al. 2011).

The most favorable climate for the initial (autumn) growth and development of rye is likely to remain at 7.0–8.3 °C with precipitation of 48 mm per month (Dzieżyc et al. 1987). Dzieżyc et al. (1987) claim that the optimum precipitation between April and July for rye grown on light soil should total 251 mm, distributed as follows: 34 mm in April, 60 mm in May, 84 mm in June, and 73 mm in July. Experimental yields of winter rye grown on suitable soils ranged from 3.19 to 5.27 t ha^−1^ (Budzyński et al. 2003). Four of the agronomic factors they analyzed significantly affected grain yield: sowing date, cultivar, NPK fertilizer, and weed control. Chemical weed control added an average 0.24 t ha^−1^, i.e., 6 % of grain yield compared to rye fields grown without chemical treatments (Rola and Domaradzki 2001). A relatively low effectiveness of chemical weed control on winter rye fields confirms that rye is highly competitive toward weeds (Deryło and Szymankiewicz 1998). An exception to this is when rye is grown on the same land in two or more consecutive years, when it is at risk of being infested by common windgrass *Apera spica-venti* L. and noxious dicotyledonous weeds. Such limited rotations require application of herbicides, which can improve grain yield by about 15 % (Budzyński 2001). In our experiment, the dramatic effect on yield of a change in seed rate and the application of herbicide almost coincided with a step-like change in temperature which has been more widely identified (Reid et al. 2015) but which was not significantly influential.

Our study clearly shows changing cultivation and phenology over the long-term, but strongly suggests that natural factors, especially temperature, continue to play a key role in understanding crop phenology, which is important from both an agronomic and medical perspective.
